# Liddle’s-like syndrome associated with nephrotic syndrome secondary to membranous nephropathy: the first case report

**DOI:** 10.1186/s12882-018-0916-3

**Published:** 2018-05-23

**Authors:** Eriko Yamaguchi, Kazuhiro Yoshikawa, Izaya Nakaya, Karen Kato, Yoshikazu Miyasato, Terumasa Nakagawa, Yutaka Kakizoe, Masashi Mukoyama, Jun Soma

**Affiliations:** 1 Department of Nephrology and Rheumatology, Iwate Prefectural Central Hospital, 1-4-1, Morioka, 020-0066 Japan; 2 Department of Nephrology, Kumamoto University School of Medicine, Honjyo 1-1-1, Chuo-Ku, Kumamoto, 860-8556 Japan

**Keywords:** Liddle’s syndrome, Epithelial sodium channel, Nephrotic syndrome, Membranous nephropathy, Urinary plasmin

## Abstract

**Background:**

Liddle’s syndrome is a rare monogenic form of hypertension caused by truncating or missense mutations in the C termini of the epithelial sodium channel (ENaC) β or γ subunits. Patients with this syndrome present with early onset of hypertension, hypokalemia, metabolic alkalosis, hyporeninemia and hypoaldosteronism, and a potassium-sparing diuretics (triamterene or amiloride) can drastically improves the disease condition. Although elderly patients having these characteristics were considered to have Liddle’s syndrome or Liddle’s-like syndrome, no previous report has indicated that Liddle’s-like syndrome could be caused by nephrotic syndrome of primary glomerular disease, which is characterized by urinary excretion of > 3 g of protein/day plus edema and hypoalbuminemia, or has explained how the activity function of ENaC could be affected in the setting of high proteinuria.

**Case presentation:**

A 65-year-old Japanese man presented with nephrotic syndrome. He had no remarkable family history, but had a medical history of hypertension and hyperlipidemia. On admission, hypertension, spironolactone-resistant hypokalemia (2.43 mEq/l), hyporeninemic hypoaldosteronism, and metabolic alkalosis, which suggested Liddle’s syndrome, were observed. Treatment with triamterene together with a steroid for nephrotic syndrome resulted in rapid and remarkable effective on improvements of hypertension, hypokalemia, and edema of the lower extremities. Renal biopsy revealed membranous nephropathy (MN) as the cause of nephrotic syndrome, and advanced gastric cancer was identified on screening examination for cancers that could be associated with the development of MN. After total gastrectomy, triamterene was not required and proteinuria decreased. A mutation in the β or γ subunits of the ENaC gene was not identified.

**Conclusion:**

We reported for the first time a case of Liddle’s-like syndrome associated with nephrotic syndrome secondary to MN. Aberrant activation of ENaC was suggested transient during the period of high proteinuria, and the activation was reversible with a decrease in proteinuria.

## Background

Liddle’s syndrome, which was firstly reported by Grant Liddle and co-workers in 1963 [1], is an autosomal dominant monogenic form of salt-sensitive hypertension with hypokalemic metabolic alkalosis and hyporeninemic hypoaldosteronism in young patients [2]. It is caused by constitutive activation of the amiloride-sensitive epithelial sodium channel (ENaC) [2]. ENaC is an ion channel expressed in sodium-transporting epithelial cells such as principal cells of the collecting ducts in kidneys [3]. ENaC is composed of homologous α, β, and γ subunits that share similar structures. Gene mutations associated with Liddle’s syndrome occur in either the β or γ subunits and disturb or truncate a conserved proline-rich sequence (i.e., PY motif), leading to constitutive activation of ENaC [4, 5]. For the treatment of Liddle’s syndrome, amiloride or triamterene, which is a potassium-sparing diuretics and a direct ENaC inhibitor, is administered together with a low sodium diet in order to inhibit sodium transport through ENaC and mitigate sodium-sensitive hypertension. The effects of these medications are rapid and exceptionally good. Spironolactone, another potassium-sparing diuretics, has no therapeutic effect, because in Liddle’s syndrome ENaC is activated even under hypoaldosteronism in Liddle’s syndrome [2].

Here, we report an elderly patient with Liddle’s-like syndrome caused by nephrotic syndrome probably secondary to membranous nephropathy (MN). Although the urine from experimental nephrotic rats and patients with nephrotic syndrome was proved to activate ENaC in vitro [6, 7], there has been no actual case report on Liddle’s syndrome caused by nephrotic syndrome associated with a primary glomerular disease and on the functional changes of ENaC according to the severity of proteinuria. To our knowledge, this is the first such case in the literature. The present case suggested that ENaC activity may increase in the setting of high proteinuria and that the activity could become normal through the reduction or disappearance of urinary protein.

## Case presentation

A 65-year-old Japanese man presented with increasing edema of the lower extremities, which he noted for about one year. In a routine medical check-up two years previously, no proteinuria was identified. He had no remarkable family history, and had a medical history of hypertension and hyperlipidemia which were treated with olmesartan medoxomil, amlodipine, and pravastatin sodium. His blood pressure control was good. Two weeks before presentation, he had cerebral infarction. Although the infarction was mild, general edema was exacerbated and pleural effusion appeared. Therefore, he was transported to our hospital.

On admission, his blood pressure was 162/92 mmHg and pulse rate was regular at 80 beats/min. Marked edema of the lower extremities, articular disorder and a positive Barré’s sign, a diagnostic sign indicating a disease of the pyramidal tracts, were noted on physical examination. Urinalysis showed proteinuria of 14.3 g/day and 23.8 red blood cells/high-power field with hyaline casts. Serum creatinine, blood urea nitrogen, total protein and albumin levels were 138 mmol/dL, 7.4 mmol/L, 43 g/L and, 19 g/L, respectively. Estimated glomerular filtration rate (eGFR), which was calculated by Japanese equation for eGFR [8], was 36.0 ml/min/1.73 m^2^. Severe hypokalemia (2.43 mmol/L) was observed under the treatment with furosemide (10 mg/day) and spironolactone (50 mg/day), while the urinary potassium level was 17.6 mmol/L. Arterial gas analysis showed metabolic alkalosis (pH, 7.54; pO_2_, 64 mmHg; pCo_2_, 41 mmHg; and HCO^3−^, 35.1 mmol/L). Despite both hypoalbuminemia and the use of two diuretics, the plasma renin activity (PRA) and plasma aldosterone concentration (PAC) were suppressed to undetectable levels (Table 1). Urine cortisol was slightly increased, but adrenocorticotropic hormone, plasma cortisol and plasma 11-deoxycorticosterone levels were within the normal ranges (Table 1).

**Table 1:** Endocrinological data

	On admission	After CR of NS	Normal range
PRA	≦0.1	0.8	0.3–2.9 μg/L/h
PAC	≦0.28	1.25	0.80–4.40 nmol/L
Plasma cortisol	295	NA	171–535 nmol/L
Urine cortisol	265	NA	31–222 nmol/day
Plasma ACTH	6.7	NA	1.2–13.9 pmol/L
Plasma 11-DOC	0.0048	NA	0.0024–0.0085 nmol/L

Urine cortisol was measured using 24-h collected urine. Blood sampling was performed early in the morning

*PRA* plasma renin activity, *PAC* plasma aldosterone concentration, *ACTH* adrenocorticotropic hormone, *11-DOC* 11-deoxycorticosterone, *CR* complete remission, *NS* nephrotic syndrome, *NA* not available, *h* hour

**Fig. 1:**
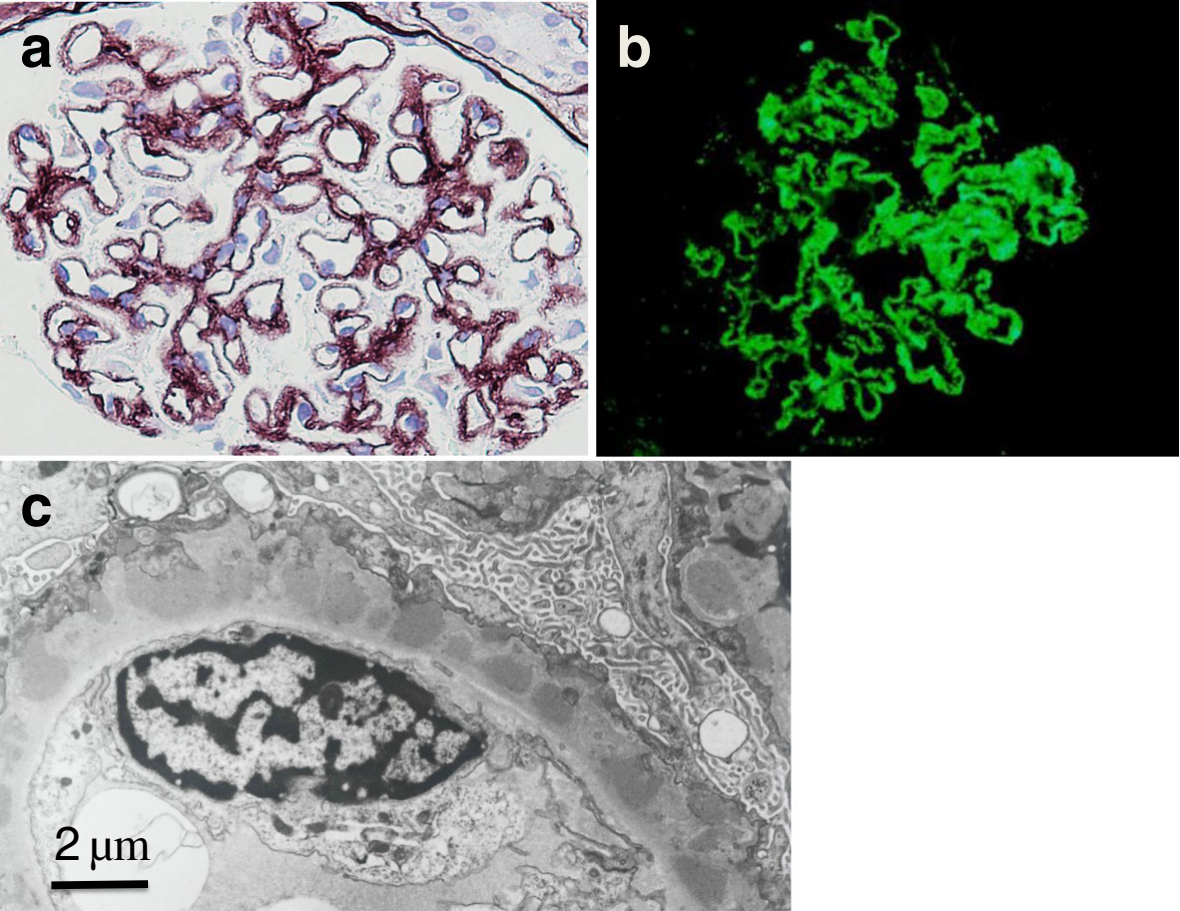
Histological findings of the patient. **a** On light-microscopy (PAM staining, × 200), diffuse thickening of the capillary wall is noted with moth-eaten appearance and spike formation. **b** On immunofluorescence examination, granular IgG deposits are markedly observed along the capillary walls. **c** On electron-microscopy, subepithelial electron-dense deposits and spikes are seen

A renal biopsy showed findings of diffuse thickening of the glomerular capillary walls with spikes and moth-eaten appearance but not mesangial or endocapillary proliferative changes (Fig. 1a). On immunofluorescence examination, marked IgG granular deposits were observed along the capillary walls (Fig. 1b). On electron microscopy, numerous subepithelial dense deposits were found. These deposits were closely approximated with intervening spikes or encircled and incorporated into the capillary walls by spikes (Fig. 1c). The interstitium was edematous and increased slightly. Foamy changes of paroxysmal tubules were patchy, but no remarkable changes of the distal tubules were noted. These finding were comparable to stage II to III MN.

The patient was first treated with oral prednisolone (30 mg/day) for MN, and then, triamterene (50 mg/day) was added after considering a diagnosis of Liddle’s-like syndrome. The dose of triamterene was increased to 100 mg/day 5 days after the start of administration. Although proteinuria did not decreased, blood pressure, serum potassium levels, and edema were remarkably improved. In parallel with these treatments, screening examinations for cancer as a possible cause of MN were conducted, and advanced gastric cancer (type V) was found. Although proteinuria of over 5 g/day was still observed, prednisolone was quickly tapered to 10 mg/day, and total gastrectomy was performed. Two weeks after the gastrectomy, proteinuria was decreased to approximately 2 g/day. As the patient began to display hypotension (systolic blood pressure < 100 mmHg), triamterene was discontinued. After the cessation of triamterene, neither hypotension nor hypokalemia was observed. Prednisolone at 10 mg/day was continuously administered. Proteinuria continued to decrease gradually and disappeared 8 months after the operation. Finally, nephrotic syndrome showed complete remission, and laboratory data showed normal serum levels, PRA, and PAC (Table 1), and no metabolic alkalosis even without triamterene treatment.

After obtaining informed consent, genetic mutations of the β and γ subunits of ENaC were assessed using a direct sequence approach as previously described [9]. However, no genetic mutation was detected in either of the subunits.

## Conclusions

Our patient presented with hypertension, hypokalemia, hyporeninemic hypoaldosteronism, and concomitant alkalosis. Liddle’s syndrome was suspected, and triamterene was administered. Its effect was exceptionally good, and both blood pressure and serum potassium levels quickly improved. The clinical symptoms, laboratory data, and good response to triamterene were suggestive of a diagnosis of Liddle’s syndrome. However, we doubted whether this diagnosis was accurate because of the high age of the patient. Actually, triamterene was not needed to treat the patient after remission of nephrotic syndrome associated with MN, which was probably secondary to gastric cancer. In addition, the subsequent evaluation for mutations in the β or γ subunits of the ENaC gene was negative [4, 5]. The present case demonstrates that high proteinuria in primary glomerular disease could activate ENaC and cause Liddle’s-like syndrome, and that the activation of ENaC could be transient and could be normalized with the decrease or disappearance of urinary protein.

Liddle’s syndrome is an autosomal dominant disease [2]; however, rare sporadic cases of the disease caused by de novo ENaC mutations have been reported [5, 7, 9]. Patients with autosomal dominant disease or sporadic mutations have the common characteristic of early onset of hypertension. In contrast, cases of Liddle’s-like syndrome have been reported in elderly patients [10–13]. Tapolyai et al. (2010) performed a cohort study of predominantly elderly patients with a mean age of 67.1 years and reported that contained 9 patients (6%) satisfied the criteria for likely Liddle’s syndrome [13]. However, a family history of the disease was not found in these elderly patients [10–13], and no testing of ENaC was performed, except one case, which had a negative result [12]. The following two possibilities for Liddle’s-like syndrome have been reported: (1) Liddle’s-like syndrome might be associated with different inherited or acquired mutations in older adults; (2) age-, polypharmacy-, or renal disease-mediated dysfunction of the epithelial sodium cannel might cause Liddle’s-like biochemical profiles [11].

The urine from puromycin aminonucleoside-induced nephrotic rats and patients with nephrotic syndrome was reported to activate ENaC in vitro [14, 15]. In these studies, aberrant presence of a soluble serine protease identified as plasmin in the urine from nephrotic rats and patients with nephrotic syndrome was confirmed. A leaky glomerular filtration barrier allows filtration of proteases or precursors of protease with the ability to activate ENaC. Plasmin is generated in tubular fluid from filtered plasminogen by an amiloride-sensitive urokinase-type plasminogen activator, and active plasmin in urine stimulates ENaC through cleavage of the γ subunit of ENaC [14]. Therefore, in the present case, it was highly suspected that increased plasmin levels in the urine might have caused aberrant activation of ENaC through the functional changes of the γ subunit of ENaC, although we did not measure the plasmin level in the patient’s urine. Furthermore, we could confirm that functional and structural changes of ENaC in nephrotic syndrome are reversible depending on the degree of proteinuria.

Although the close association of high proteinuria with the activation of ENaC were confirmed in vitro as mentioned above, it is surprising that no English report has described an actual case of Liddle’s-like syndrome associated with renal disease. Three Japanese reports were identified. One case involved polycystic kidney disease [16], one involved tubulopathy due to hypercalcemia associated with vitamin D treatment [17], and one involved diabetic nephropathy with macroalbuminemia [18]. The second case did not present with Liddle’s-like syndrome after the cessation of vitamin D treatment. Interestingly, diabetic nephropathy has been reported to be associated with increased urinary excretion of plasmin, prostansin, and urokinase and proteolytic activation of ENaC even in non-nephrotic patients [19]. However, Liddle’s-like syndrome caused by nephrotic syndrome associated with a primary glomerular disease has not been reported in Japanese as well. The present case is unexpectedly the first such case in the literature; however, it is possible that many cases similar to the present case were overlooked with regard to not only MN but also in other glomerular diseases such as minimal change nephrotic syndrome, focal segmental glomerulosclerosis, and membranoproliferative glomerulonephritis. We should consider the possibility of Liddle’s-like syndrome when treating nephrotic patients, as triamterene or amiloride (blocker of ENaC) could be a therapeutic option for resistant hypertension or edema in the acute stage of nephrotic syndrome.

In conclusion, we described the first case report of Liddle’s-like syndrome caused by nephrotic syndrome associated with MN. We believe that such cases are not very rare and many similar conditions in nephrotic syndrome might have been overlooked. We should pay more attention to ENaC activity and nephrotic syndrome by assessing PRA, PAC, serum potassium levels, and the acid-base balance. Our findings may lead to a unique and effective therapy using triamterene or amiloride for intractable hypertension or edema in nephrotic syndrome.

## Abbreviations

11-DOC: 11-deoxycorticosterone
ACTH: Adrenocorticotropic hormone
CR: Complete remission
ENaC: Epithelial sodium channel
h: Hour
MN: Membranous nephropathy
NA: Not available
NS: Nephrotic syndrome
PAC: Plasma aldosterone concentration
PRA: Plasma renin activity
